# Supplementary material for: Assessing the ecological regime and spatial spillover effects of a reclaimed mining subsided lake: A case study of the Pan'an Lake wetland in Xuzhou
Source: PLoS One. 2020 Aug 27;15(8):e0238243. doi: 10.1371/journal.pone.0238243 (PMC7451651; doi:10.1371/journal.pone.0238243)
Supplement: Attachment — Submitted filename: manuscript-revised.docx [file pone.0238243.s002.docx]

**Assessing the Ecologic Regime and its Spatial Spillover Effect of a Reclaimed Mining Subsided Lake: A Case Study of the Pan'an Lake Wetland in Xuzhou**

Jiaxing Xu^1^, Pengcheng Yin^2, 3^, Wenmin Hu^1,^*, Lili Fu^4^ and Hua Zhao^3^

^1^	The National and Local Joint Engineering Laboratory of Internet Applied Technology on Mines, China University of Mining and Technology, Xuzhou 221116, China;

^2^	Natural Resources and Planning Bureau of Xuzhou, Xuzhou, 221018, China;

^3^	School of Environmental Science and Spatial Informatics, China University of Mining and Technology, Xuzhou, 221116, China;

^4^	Jiangsu Zenith Geo-Informatics Co., Ltd, Xuzhou 221116, China;

*	Correspondence: Dr. Wenmin Hu

E-mail: [huwm@cumt.edu.cn](mailto:huwm@cumt.edu.cn) (WM H)

**Abstract**

Coal mining subsided lake is a special surface water body formed after coal mining in the plains of eastern China. Transforming mining subsided lakes into artificial wetland parks is an effective ecological restoration approach that has been attempted in China. While many studies focused on evaluating the ecosystem service value and monitoring water pollution in coal mining subsidence lakes, little attention has been paid to the changes in the ecologic regime and the ecological spillover effect before and after the reclamation of the mining area. This paper took the Pan'an Lake Wetland, an artificial wetland derived from restoration of a mining subsided lake in Jiawang District, Xuzhou, as a case area. The changes of ecologic regime in the mining subsidence area before and after land reclamation and their spatial spillover effect on land prices were assessed based on remote sensing, GIS raster calculation and geostatistical methods. The results showed that the ecosystem service value and ecological storage capacity have changed significantly after the mining subsided lake was transformed into an artificial wetland and the wetland ecosystem has been developing well and has proposed a significant positive spillover effect on surrounding land prices. From 2008 to 2017, the service functions of the artificial wetland ecosystem in Pan'an Lake increased by 81.95%, and the ecological storage capacity increased from RMB 6,754 yuan/hm^2^ in 2008 to RMB 12,289 yuan/hm^2^ in 2017. The average impact intensity of the Pan'an Lake Artificial Wetland on the spillover effect of surrounding residential land prices was RMB 195.18 yuan/m^2^ and the total spillover value of the planned residential land in the study area was RMB 805,422,100 yuan. This work provides useful guidance and reference for evaluating the economic feasibility of land reclamation planning and ecological restoration in mining subsidence areas.

**1. Introduction**

Ecosystem in mining area are a complex system, which comes into being due to the interaction between the natural environment system and the social environment system dominated by the development and utilization of mineral resources within the mining area [1-2]. The long-term, large-scale and high-intensity mineral resource development activities have inevitably destroyed the geological conditions of the native deposits, giving rise to surface subsidence, soil pollution and plant destruction as well as changes in hydrothermal structures and damage to the ecological environment, so that the whole ecosystem is extremely vulnerable [3-6]. It was estimated that the average land collapse rate per 10,000 tons of coal mining would be 0.20-0.33 hm^2^ in China [7]. It is predicted that the cumulative subsidence area due to coal resource exploitation will be more than 1.5 million hm^2^ in China by 2020, which will still increase at a rate of 30,000 to 47,000 hm^2^ every year [6]. Therefore, one of the important issues in the current studies on sustainable development of mining areas is how to scientifically carry out comprehensive rectification and utilization of subsidence lands in the mining areas.

Mining subsidence area in the North China Plain is a special mining area with high groundwater levels. Because the groundwater level is relatively shallow, the subsidence area may be flooded easily, finally forming a large waterlogged area. As the submerged area is expanding due to coal mining, the permanent ponding area may become a pond or even a lake, namely, a coal-mining subsidence lake, which may form a wetland ecosystem in a long run [8]. As the balance of water system is broken, the water body may be polluted by mine water, leaching water from waste heap, etc., the landscape is destroyed and land productivity is reduced. So, these places have very little land-use effect on agricultural or urban development and are considered as economic “scars” [9-12]. As an important link in the production and construction of the mines, land reclamation is an important means to minimize environmental damage caused by mining and restore local ecological balance [13]. In recent years, it has been an attempt to explore the ecological restoration model of mining areas in China by reclaiming mining subsidence areas into artificial wetland parks according to local conditions with land reclamation techniques [14]. The rectification of the mining subsidence lake area is a difficult point in ecological restoration and resource utilization in mining areas. The successful rectification of the mining subsidence lake is subject to the ecosystem restoration conditions and economic benefits in the mining areas after the reclamation. Therefore, it is of great practical and theoretical significances to evaluate and research the ecosystem in the reclamation area for the implementation of land reclamation and ecological restoration projects in the mining area.

Wetlands offer many essential ecosystem services to human being. They have such service functions as supply, regulation, culture, support and so forth, including climatic regulation, water purification, entertainment, biodiversity, etc. [15-16]. Wang et al. [17] constructed an ecological evaluation system, which was composed of the values in products, regulation services and cultural services of the ecosystem, and evaluated the changes in total amount of the Nansi Lake Wetland ecosystem by using remote sensing image data from 1985 to 2017. In recent years, as ecological restoration and rectification went further, the rectified wetlands could significantly improve the wetland ecosystems, increased their ecological storage capabilities and produced positive effects on service value of the wetland ecosystem and its surrounding economic benefits. Throughout the mining cycle, Demirbugan [18] analyzed the net benefits of ecosystem services and change profile emerged during the mining and rehabilitation process in the Soma coal region located in the western part of Turkey. Sun et al. [12] showed that converting a mining subsided lake to a restored wetland through land reclamation and ecological restoration can provide economic and ecological benefits via the return of their ecological services and functions. Wang et al. [19] proposed an eco-environmental benefit evaluation method based on land-use changes and evaluated the changes in the eco-environmental benefits during the reclamation of the abandoned land in Fushun Xilutian Mine. The results showed that three major eco-environmental functions (soil formation and protection, biodiversity protection and air regulation) improved the most after the reclamation, which accounted for about half of the total eco-environment benefits.

At present, the studies on the ecological environment of mining subsidence lakes mainly focus on evaluating ecosystem service values, monitoring water pollution and nutrition status, restoring ecological landscape, etc. [20-23]. However, there are few studies focusing on the ecological restoration conditions and their ecological environment effects before and after the reclamation. The land reclamation in coal mining areas is still dominated by scattered projects and guided by economic benefits, such as increasing cultivated land or linking reclamation and utilization, to determine the reclamation area, but less attention is paid to ecological and environmental effects. In addition, ecological environment service, as a public service, presents an obvious spatial spillover [24]. However, the land reclamation of mining areas lays stress on economic benefits, but ignores environmental externalities, so that the policies pay limited attention to the environment and the entire ecosystem, which greatly weakens the activeness for reclamation [12].

With the Pan'an Lake Wetland as the study area, this paper combined the remote sensing, GIS raster calculation and geostatistical methods to assess the ecosystem changes and their spatial spillover effect in the mining subsidence area before and after land reclamation, so as to provide important technical support for assessing land reclamation and ecological restoration effect in mining areas and also offer scientific methods for determining reclamation models in the mining area.

2. Materials and Methods
   2.1. **Study Area**

The Pan'an Lake Wetland is an artificial wetland derived from restoration of a mining subsided lake in the southwest of Jiawang District, Xuzhou City (117º19’57’’-117º26’15’’E, 34º19’17’’-34º23’33’’N). Due to resource exhaustion, coal-mining subsidence, ecological degradation and so on, the economic level of the Jiawang Mining Area, where the Pan'an Lake Wetland lies, is lower than that in main urban area of Xuzhou City and is a depression for economic development of Xuzhou City. Since 2010, Jiawang District implemented land reclamation and ecological restoration in the mining subsidence area. As the first wetland park for ecological restoration in a coal-mining subsidence area countrywide, the Pan'an Lake Wetland was constructed in a four-in-one model integrating “basic farmland rectification, coal-mining subsidence reclamation, ecological environment restoration and wetland landscape development”. By 2013, the national 4A Ecological Wetland Park, including a water area of 9.21km^2^ and an overall area of 52.89 km^2^, had been completed. The location of the study area is shown in Fig 1.

**Fig 1. Location and image map of the study area.**

2.2. **Data sources and processing**

The data in this paper were mainly from two phases of multi-temporal remote sensing images, including QuickBird high-resolution images in 2008 and 2017, 1:10,000 topographic map of Jiawang District, land-use diagram in 2017, and land price survey data during 2008-2017. Firstly, the topographic map of Jiawang district was used to accurately register the remote sensing images in the ERDAS software; the Random Forest (RF) classification was combined with visual interpretation to obtain the land-use data before and after the reclamation in the study area. Based on the characteristics of the study area, the land-use types in the study area were divided into 6 types: cultivated land, woodland, grassland, transportation land, general water bodies (including rivers and ponds), subsided water bodies, urban/rural construction land and industrial/mining construction land, of which the Kappa coefficients were all above 0.95 and met the data accuracy requirements of this study. According to the land-price sample survey tables in Jiawang District in 2008 and 2017, a total of 47 residential land price samples in 2008 and 65 residential land price samples in 2017 were collected, for which spatial interpolation was performed using GIS and geostatistics methods.

2.3. **Methodology**

Before and after the reclamation in the study area, the complex ecosystem in the ecologically degraded mining area has been transformed into a land-water composite wetland ecosystem after many years of ecological restoration and environmental rectification. In order to link the abstract ecological concept with specific economic benefits to better evaluate and measure the ecological restoration conditions in the study area, this dissertation made relevant analysis from three aspects: ecosystem service value, ecological storage capacity and spillover effect.

2.3.1. **Ecological service value evaluation model**

The ecosystem service value evaluation model proposed by Costanza et al. [25] was adopted in this paper.

$ESV=\sum A_{i}\times VC_{i}$ (1)

where *ESV* is the ecosystem service value (RMB), *A_i_* is the area of Type *i* land use (hm^2^) and *VC_i_* is the ecosystem service value coefficient of Type *i* land use (RMB/hm^2^·a). As a kind of special surface water in the mining subsidence area, the subsided water area makes the mining area evolving from a single farmland terrestrial ecosystem to a wetland / aquatic ecosystem with the functions of climatic regulation, water conservation and raw material supply; meanwhile, it is susceptible to various pollutions, such as "industrial wastes", pesticides, fertilizers, domestic sewage and other pollutants, around the mining area and is short of soil formation and protection, entertainment and cultural functions, etc. The ecosystem service value coefficients of natural water and collapsed water per unit area are shown in Table 1 [3]. At the same time, the service value system per unit area of China's ecosystem formulated by Xie et al. [26] and the value system of mining cities, towns and industrial ecosystems estimated by Zhang et al. [27] were used and the value coefficient of ecological services in the study area is obtained (Table 2).

**Table 1. Comparison of ecosystem service values per unit area between natural waterbody and subsided waterbody.**

| Ecosystem Service | Natural Waterbody | | Subsided Waterbody | |
| --- | --- | --- | --- | --- |
|  | Ecosystem Service | Value（yuan/hm^2^） | Ecosystem Service | Value（yuan/hm^2^） |
| Climate regulation | √ | 407 | √ | 407 |
| water conservation | √ | 18033.2 | √ | 4508.3 |
| Soil formation and conservation | √ | 8.8 | × | 0 |
| waste treatment | √ | 16086.6 | √ | 4021.65 |
| Biodiversity maintenance | √ | 2203.3 | √ | 1101.65 |
| Food production | √ | 88.5 | √ | 88.5 |
| Raw material | √ | 8.8 | √ | 8.8 |
| Recreation and culture | √ | 3840.2 | × | 0 |
| Total |  | 40676.4 |  | 10135.9 |

**Table 2. Value coefficients of service functions of different terrestrial ecosystems in the Pan'an Lake Wetland.**

| Ecosystem Type | Cultivated land | Woodland | Grassland | Natural waterbody | Subsided waterbody | Industry/  transportation | Urban |
| --- | --- | --- | --- | --- | --- | --- | --- |
| Value Coefficients | 6114.3* | 19334* | 6406.5* | 40676.4* | 10135.9* | -5372.1* | -1760.5* |

*Unit: yuan·hm^-2^·a^-1^

2.3.2. **Ecological storage model**

Ecological storage state (ESS) describes the correlation between land-use changes and their ecological responses. It is a comprehensive expression of ecological changes caused by land-use quantity, quality, type and distribution determined by possible natural or human activities in the past and the future. The ecological storage state reflects the level of comprehensive ecological storage in the region and is determined by one or more dominant land-use types in this region. The formula is as follows [28]:

$ESS=\frac{1}{h}\cdot\frac{\sum_{i=1}^{n} ESV_{i}}{\sum_{i=1}^{n} A_{i}}$ (2)

Where, *ESS* is the ecological storage state in the mining area, *h* is the estimated number of years corresponding to the ecological storage state in the mining area (usually, *h*=1 for the ecological storage state of a certain year), *ESV_i_* is the ecological service value corresponding to the ecosystem Type *i*, and *A_i_* is the area of ecosystem Type *i*.

2.3.3. **Spillover effect measurement model**

Land prices are jointly affected by various factors. However, except for land reclamation and ecological restoration projects, there are no other major projects or factors affecting land prices in the study area. Therefore, as long as the average growth value (general changes) of land prices in the study area is separated, it is possible to analyze the spillover effect of the subsided lake wetland rectification on surrounding land prices [29]. Calculation steps of spillover effect is shown as follows (Fig 2).

**Fig 2. Spillover effect model framework of land price for the Pan'an Lake Wetland.**

1) Calculate the average rise in land prices in the subsided area before and after land reclamation and study the general changes in regional land prices.

2) Calculate the rise in land prices around the study area before and after land reclamation and study the changes in the surrounding land prices.

3) The amplitude and extent that the rise in the land price around the study area is higher than the average rise of regional land price are taken as the strength and scope of the subsided lake wetland on the spillover effect of surrounding land price.

Based on the above ideas, this paper proposes the following equation for calculating the spillover effect of subsided lake wetlands on surrounding land prices:

$SEV=\sum\left(Lp_{ij}^{\alpha}-Lp_{ij}^{\beta}-Lp_{ij}^{\beta}\cdot r_{i}\right)\times S_{ij}$ (3)

where *SEV* is the value of the collapsed lake wetland on the spillover effect of the surrounding land value and is expressed in land price appreciation; $Lp_{ij}^{\alpha}$ and $Lp_{ij}^{\beta}$ are separately the land prices of Plot *j* of Type *i* land after and before land reclamation; *r_i_* is the average rise of Type *i* land prices in the area of the subsided lake wetland; *S_ij_* is the area of Plot *j* of Type *i* land in the subsided area.

3. Results and Analysis

**3.1. Changes in land use and ecosystem service value**

According to the interpretation results of remote sensing images in 2008 and 2017, the landscape types and their changes before and after reclamation in the study area were shown in Fig 3 and Table 3. As can be seen from Table 3, there are 7 types of land use in the study area, among which cultivated land is an important part of the land use structure. In 2008, the cultivated land accounted for more than half of the total area. Along with the rehabilitation of the ecological environment in the Pan'an Lake Wetland Park, the area of cultivated land was reduced to 39.27% from original 54.98%. Next, the proportions of woodland and grassland increased from 0.63% and 2.43% to 8.47% and 9.39%, respectively. The subsided water bodies were transformed into lake wetlands through land reclamation and the area increased by 152.15hm^2^, an increase of 16.55%. With the depletion of coal resources and land reclamation planning in Jiawang District, coal mines were closed, coal-mining villages were relocated, and abandoned industrial and mining sites were gradually removed, so that industrial/mining and construction land decreased by 12.14%.

**Fig 3. Landscape pattern before and after land reclamation in the Pan'an Lake subsidence area.** (a) landscape types in 2008; (b) landscape types in 2017.

**Table 3. Landscape structure changes in the Pan'an Lake subsidence area before and after reclamation.**

| Landscape Type | Before land reclamation | | After land reclamation | |
| --- | --- | --- | --- | --- |
|  | Area/hm^2^ | Percentage /% | Area/hm^2^ | Percentage /% |
| Cultivated land | 2918.97 | 54.98 | 2084.89 | 39.27 |
| Woodland | 33.62 | 0.63 | 449.84 | 8.47 |
| Grassland | 128.75 | 2.42 | 498.67 | 9.39 |
| Transportation land | 209.69 | 3.95 | 223.85 | 4.22 |
| Natural Waterbody | 336.59 | 6.34 | 1071.63 | 20.18 |
| Urban/ Industry/Mining area | 1098.91 | 20.70 | 980.54 | 18.47 |
| Subsided Waterbody | 582.89 | 10.98 | 0.00 | 0.00 |

As shown in Table 3, the value of ecosystem services in the study area increased by 81.95%. Among them, the value of individual ecological services of forest land, grassland and water body increased by 1238.05%, 287.28% and 218.38%, respectively. The main reason is that the coal-mining subsidence land has been reclaimed into large areas of artificial wetland, woodland, grassland, etc. during the land reclamation process of the mining area, so that their ecosystem service values have increased. The ecosystem service values of urban, industrial land, cultivated land, and subsided water bodies were decreasing, mainly due to the construction of artificial wetland projects, the removal of coal-mining villages and the withdrawal of industrial and mining land in the mining subsidence area, the cultivated land and the land for villages and industrial and mining activities have gradually decreased in the study area and their ecological service values have declined continuously. After ecological restoration, the subsided water bodies have been transformed into lake wetlands. Among 17 individual ecosystem service functions, the functions of atmospheric regulation, water conservation, entertainment, waste treatment, food production and biodiversity maintenance are in an upward trend, mainly because the area of water bodies increased substantially during coal mining and land reclamation in subsidence areas.

**Table 4. Changes of EVS and value structure in the Pan'an Lake subsidence area before and after reclamation.**

| Landscape Type | Before land reclamation | | After land reclamation | | Changes of ESV | |
| --- | --- | --- | --- | --- | --- | --- |
|  | ESV | Contribution ratio(%) | ESV | Contribution ratio(%) | ESV | Change rate(%) |
| Cultivated land | 1784.75 | 49.77 | 1274.77 | 19.54 | -509.98 | -28.57 |
| Woodland | 65 | 1.81 | 869.73 | 13.33 | 804.73 | 1238.05 |
| Grassland | 82.49 | 2.3 | 319.47 | 4.9 | 236.98 | 287.28 |
| Transportation land | -112.65 | -3.14 | -128.31 | -1.97 | -15.66 | 13.90 |
| Natural Waterbody | 1369.12 | 38.18 | 4359.02 | 66.81 | 2989.9 | 218.38 |
| Urban/Industry/Mining area | -193.46 | -5.39 | -169.98 | -2.61 | 23.48 | -12.14 |
| Subsided Waterbody | 590.81 | 16.48 | 0 | 0 | -590.81 | -100.00 |
| Total | 3586.06 | 100 | 6524.69 | 100 | 2938.63 | 81.95 |

**3.2. Change of ecological storage state**

It can be seen from Table 5 that the ecological storage capacities was RMB 6,054 yuan/hm^2^ in 2008 and RMB 12,289 yuan/hm^2^ in 2017, of which the annual conversion rate was 6.15%. The results showed that the entire ecosystem was developing positively from 2008 to 2017. The active ecological storage transformation mainly benefited from land reclamation and ecological restoration projects in the mining area. Through land reclamation, the landscape pattern and ecological environment have been changed in the mining subsidence area, the land damaged by mining has been reclaimed into cultivated land, woodland and grassland, and coal-mining subsidence (ponding) area has been reclaimed into artificial wetlands, fish ponds, tourism and sightseeing land, etc., which has greatly improved the regional landscape diversity and certainly promoted the regional ecological function. However, the landscape planning has greatly increased the area of water bodies in the study area, but sacrificed other land, which proposed a certain inhibition for landscape diversity.

**Table 5. Ecological storage state of the Pan'an Lake Wetland before and after reclamation.**

| Statistics type  Year | Ecological storage state | Conversion quantity of ecological storage(2008-2017) | Conversion percent of ecological storage(2008-2017) |
| --- | --- | --- | --- |
| 2008 | 0.6754 | 0.5535 | 6.15 |
| 2017 | 1.2289 |  |  |

**3.3. Measurement of spillover effect of Subsided Lake Wetland on surrounding land prices**

In this paper, the residential land prices were sampled in Jiawang District and the surrounding area of the Pan'an Lake Wetland in Xuzhou City and the sample data were from land-price sample survey tables in Jiawang District in 2008 and 2017. A total of 47 residential land-price samples in 2008 and 61 residential samples in 2017 were collected on the same evaluation base date. Since there was less residential land around the Pan'an Lake Mining Subsidence Area in 2008, some sampling points were replaced by industrial land prices in the same year. The distribution of sample points of land price is shown in Fig 4. The normal distribution test and spatial autocorrelation analysis were performed for two sets of samples and the results showed that the collected sample land prices presented good overall distribution conditions and significant spatial positive correlations and could be used for subsequent analysis.

**Fig 4. Sample distribution of residential land price in the Pan'an Lake subsidence area.** (a) sample distribution of residential land price in 2008; (b) sample distribution of residential land price in 2017.

The cross-validation method was adopted to select the optimal model of Kriging spatial interpolation and the geostatistical software GS + fitting parameters were used to provide the basis for checking and analyzing the variation function structure [29-31]. The spatial interpolation was performed for residential land price samples in 2008 and 2017 and 15m×15m grid cells were determined according to the graded grid division method of urban land to generate the curve of residential land prices before and after the reclamation (Fig 5).

**Fig 5. Surfaces of residential land prices in the Pan'an Lake subsidence area in 2008 and 2017.** (a) the surface of residential land prices in 2008; (b) the surface of residential land prices in 2017.

Thus, the following formula was used to calculate the average rise of residential land price *△LP* in the study area:

$\Delta LP=\frac{LP_{2017}-LP_{2008}}{LP_{2008}}\times100\%$ (4)

where *LP_2008_* and *LP_2017_* are the corresponding land price levels in 2008 and 2017, respectively. It is calculated that the average rise of residential land prices in the study area should be 176.81% during 2008-2017.

The land-price grid data in 2017 is subtracted by the land-price curve data in 2008 and then the average rise in land price in the study area. And then, the area greater than 0 around the Pan'an Lake Artificial Wetland can be considered as the range that the land reclamation poses an effect on surrounding residential land prices (Fig 6). It can be seen in Fig 6 that the residential land prices are in a rising trend toward the center of the study area and the rise is significantly higher than that of the evaluated land price in the study area. It shows that the implementation of the land reclamation and ecological restoration projects in the Pan'an Lake Wetland has a significant impact on land prices in and around the study area, namely, the spillover effect of land price appreciation has been generated.

**Fig 6. Impact strength and range of spillover effects on residential land prices in the Pan'an Lake Wetland.**

Thus, the spatial overlay analysis is performed according to the planning drawing for the Pan'an Lake Wetland Park and the affected area in Jiawang District and the planned residential land area in the study area has been determined to be 412.65 hm^2^, as shown in Fig 6. According to the spillover effect measurement model (eq. (3)), the spillover value of the Pan'an Lake Wetland to residential land in the study area should be RMB 805,422,100 yuan using the Raster calculation in ArcGIS 10.2 software.

4. Discussion

**4.1. Changes in ecological environment before and after the rectification of the subsidence area**

The results of this study show that the ecological service value and ecological storage capacity of the Pan'an Lake Wetland change significantly before and after the reclamation. From 2008 to 2017, the ecosystem service function in the area where the Pan'an Lake Wetland was located increased by 81.95%, and the ecological storage capacity increased from RMB 6,754 yuan/hm^2^ in 2008 to RMB 12,289 yuan/hm^2^ in 2017, an increase of about 1.8 times. Throughout the whole life cycle, mining activities had a negative impact on the ecological environment of the mining area, including production, transportation and processing [32-33]. Jiawang District, a national resource-exhausted city listed in 2011, was a century-old coal city where 360 million tons of raw coal had been cumulatively produced. Long-term and large-scale coal mining has destroyed arable land, caused ground subsidence, ponding and soil and water pollution and posed significant negative effects on the ecological environment of the mining area [34-38]. Since 2010, Jiawang District began to carry out land reclamation and ecological restoration in the coal mining area. As the first wetland park by land reclamation and ecological restoration for coal mine subsidence area in China, the Pan'an Lake Wetland has been constructed as per the four-in-one model integrating " basic farmland rectification, coal-mining subsidence reclamation, ecological environment restoration and wetland landscape development” and the constructed wetland covers about 10 square kilometers [39-40]. Through land reclamation and ecological restoration, the landscape pattern and ecological environment in the mining subsidence area have changed, and such ecological functions as atmospheric regulation, water conservation, entertainment, waste treatment, food production and biodiversity protection are on the rise [19]. The evaluation results are basically consistent with the actual situation, which indicates certain feasibility of the evaluation method.

**4.2. Spillover effect of ecological rehabilitation on land prices in mining subsidence area**

In this study, geostatistics and GIS raster calculation methods were used to generate continuous land-price curves according to the spatial interpolation method on the basis of land price samples obtained before and after ecological restoration on the same reference day, and the contour analysis was performed for the land-price curves to study the changes of regional land prices before and after the ecological restoration in coal-mining subsidence lake [41-42]. As the price of residential land was more susceptive to the improvement of the ecological environment, the sample points of residential land prices before and after the reclamation were selected for calculation and analysis. The results show that the surrounding land-price rise after the ecological restoration in the Pan'an Lake Wetland was significantly higher than the average rise of the land price in this region and the land prices were in the rising trend toward the wetland, which indicated that the implementation of land reclamation and ecological restoration projects in the mining subsidence area has produced the spillover effect of the added land values. It was calculated that the spillover value of the Pan'an Lake Wetland to residential land in the study area was RMB 805,422,100 yuan. The results indicate that the model and method constructed in this study can be used to analyze the spillover effect of ecological environment restoration in the reclamation mining area and as a case to analyze the economic benefits of reclamation in other mining subsidence areas.

**4.3. Uncertainty analysis of research results**

This paper adopted the value evaluation model of ecosystem services proposed by Costanza et al. [25] and corrected the corresponding ecological service value equivalents in combination with actual situation of the mining area, and calculated the ecological service value and its changing trend in the study area. The evaluation results of this model may be less accurate in some regions. However, a large number of studies have proved that the method is applicable to the evaluation of ecosystem services in many regions of China when the equivalent correction coefficient of ecological service value by Xie is adopted [43-45]. In addition, because many factors may affect land prices, e.g. location, supply and demand, housing price, ecological environment, etc. [46-48], the average growth of land price in the region is only used to eliminate the impact of these factors in this dissertation, which may pose a certain degree of uncertainty. Moreover, when the natural increase factor in the artificial wetland price of the mining subsidence lake is eliminated, the effect of the artificial wetland on the land price has not been considered, which may make the calculated value of the spillover effect to be on the low side. Due to the complexity and particularity of the research issues, these uncertainties and deficiencies will be further corrected and improved in future studies.

5. Conclusions

Based on remote sensing, GIS raster calculation and geostatistical methods and with the coal-mining subsided lake in the Pan'an Lake Wetland (Jiawang, Xuzhou) as a study case, this paper analyzed the restoration conditions and changes of the ecosystem before and after the reclamation in the mining subsidence area. The main conclusions are as follows:

(1) After land reclamation and ecological restoration, the ecosystem service value and ecological storage capacity in the study area have changed significantly and the entire ecosystem has been actively and positively developing with a good overall condition. From 2008 to 2017, the ecosystem service functions increased by 81.95% in the constructed Pan'an Lake Wetland, and the ecological storage capacity increased from RMB 6,754 yuan/hm^2^ in 2008 to RMB 12,289 yuan/hm^2^ in 2017, with an annual conversion rate of 6.15%.

(2) After land reclamation in the mining subsidence area of Pan'an Lake, the surrounding land prices have produced a significant positive spillover effect. The maximum intensity of the spillover effect of the residential land price in the study area is RMB 354.43 yuan/m^2^, the average value of the impact intensity is RMB 195.18 yuan/m^2^, and the total spillover value of the planned residential land in the study area was RMB 805,422,100 yuan. The model and method constructed in this study can be used to analyze the spillover effect of ecological environment restoration in the reclamation area and as a case to analyze the economic benefits of reclamation in other mining subsidence areas.

(3) The research results can offer useful guidance for future studies on land reclamation planning, ecological restoration and value performance in the mining subsidence area. However, due to the particularity of the research subject and the complexity of the research issues, there are still some deficiencies in this study. It should be further revised and improved in future studies.

Acknowledgments

We are very grateful to Jun Liang and Yanhui Zhao for their assistance with data on residential land prices and remote sensing images, Yiyan Zhang for assistance with statistical analyses. We also thank the editor and anonymous reviewers for their insightful comments on the manuscript.

References

1. Mishra PP. Coal mining and rural livelihoods: case of the Ib Valley Coalfeld, Orissa. Econ. Polit. Wkly. 2009; 44: 117–123.
2. Karan SK, Samadder SR, Maiti, S.K. Assessment of the capability of remote sensing and GIS techniques for monitoring reclamation success in coalmine degraded lands. J. Environ. Manag. 2016; 182: 272–283.
3. Bian ZF, Lu QQ. Ecological effects analysis of land use change in coal mining area based on ecosystem service valuing: a case study in Jiawang. Environ. Earth. Sci. 2013; 68(6): 1619–1630.
4. Antwi EK, Boakye-Danquah J, Asabere SB, Takeuchi K, Wiegleb G. Land cover transformation in two post-mining landscapes subjected to different ages of reclamation since dumping of spoils. SpringerPlus. 2014; 3(1): 702.
5. Blachowski J. Application of GIS spatial regression methods in assessment of land subsidence in complicated mining conditions: case study of the Walbrzych coal mine (SW Poland). Nat. Hazards. 2016; 84(2): 997–1014.
6. Xu JX, Zhao H, Yin PC, Wu LX, Li G. Landscape ecological quality assessment and its dynamic change in coal mining area: a case study of Peixian. Environ. Earth Sci. 2019; 78: 708.
7. Hu ZQ, Xiao W. Optimization of concurrent mining and reclamation plans for single coal seam: a case study in northern Anhui, China. Environ. Earth Sci. 2012; 68(5): 1247–1254
8. Krodkiewska M, Królczyk A. Impact of environmental conditions on bottom oligochaete communities in subsidence ponds (The Silesian Upland, Southern Poland). Int. Rev. Hydrobiol. 2011; 96(1): 48–57.
9. Li F, Liu X, Zhao D, Wang B, Jin J, Hu D. Evaluating and modeling ecosystem service loss of coal mining: a case study of Mentougou district of Beijing, China. Ecol. Complex. 2011; 8(2): 139.
10. Stojiljkovic E, Grozdanovic M, Marjanovic D. Impact of the underground coal mining on the environment. Acta Montan. Slovaca. 2014; 19(1): 6.
11. Pandey B, Agrawal M, Singh S. Coal mining activities change plant community structure due to air pollution and soil degradation. Ecotoxicology. 2014; 23(8): 1474–1483.
12. Sun JF, Yuan XZ, Liu H, Liu GD, Zhang GX. Energy evaluation of a swamp dike-pond complex: A new ecological restoration mode of coal-mining subsidence areas in China. Ecol. Indic. 2019; 107: 105660. (doi: [10.1016/j.ecolind.2019.105660](https://doi.org/10.1016/j.ecolind.2019.105660))
13. Xiao W, Hu ZQ, Chugh YP, Zhao YL. Dynamic subsidence simulation and topsoil removal strategy in high groundwater table and underground coal mining area: a case study in Shandong Province. Int. J. Min. Reclam. Environ. 2013; 28(4): 250–263.
14. Wang LJ, Wang LC, Yin PC, Cui HY, Liang LG, Wang ZB. Value assessment of artificial wetland derived from mining subsided lake: a case study of Jiuli Lake Wetland in Xuzhou. Sustainability 2017; 9: 1860.
15. Adekola O, Mitchell G. The Niger Delta wetlands: Threats to ecosystem services, their importance to dependent communities and possible management measures. International Journal of Biodiversity Science Ecosystem Services and Management. 2011; 7(1): 50–68.
16. Malekmohammadi B, Jahanishakib F. Vulnerability assessment of wetland landscape ecosystem services using driver-pressure-state-impact-response (DPSIR) model. Ecol. Indic. 2017; 82: 293–303.
17. Wang F, Zhang SL, Hou HP, Yang YJ, Gong YL. Assessing the Changes of Ecosystem Services in the Nansi Lake Wetland, China. Water. 2019; 11: 788.
18. Demirbugan A. Changes in ecosystem service benefit in Soma lignite region of Turkey. Resour. Policy. 2019; 64: 101522. (doi: 10.1016/j.resourpol.2019.101522)
19. Wang J, Wei ZY, Wang QB. Evaluating the eco-environment benefit of land reclamation in the dump of an opencast coal mine. Chem. Ecol. 2017; 33(7): 607–624.
20. Zhang F, Yushanjiang A, Jing Y. Assessing and predicting changes of the ecosystem service values based on land use/cover change in Ebinur Lake Wetland National Nature Reserve, Xinjiang, China. Sci. Total Environ. 2019; 656: 1133–1144.
21. Lu JZ, Lu HW, Lei KW, Wang WP, Guan YL. Trace metal element pollution of soil and water resources caused by small-scale metallic ore mining activities: a case study from a sphalerite mine in North China. Environ. Sci. Pollut. R. 2019; 26(6): 24630–24644.
22. Malaviya SM, Munsi M, Oinam G, Joshi PK. Landscape approach for quantifying land use land cover change (1972–2006) and habitat diversity in a mining area in Central India (Bokaro, Jharkhand). Environ. Monit. Assess. 2010; 170(1-4): 215–229.
23. Xu JX, Zhao H, Yin PC, Bu NS, Li G. Impact of Underground Coal Mining on Regional Landscape Pattern Change Based on Life Cycle: A Case Study in Peixian, China. Pol. J. Environ. Stud. 2019; 28(6): 4455–4465.
24. Wang SJ, Huang YY, Zhou YQ. Spatial spillover effect and driving forces of carbon emission intensity at the city level in China. J. Geogr. Sci. 2019; 29(1): 231–252.
25. Costanza R, d’Arge R, De Groot R, Farber S, Grasso M, Hannon B, et al. The value of the world’s ecosystem services and natural capital. Nature. 1997; 387: 253-260.
26. Xie G, Cao S, Xiao Y, Pei X, Bai Y, Li W, et al. Ecosystem Service Evaluation. In Contemporary Ecology Research in China; Springer: Berlin/Heidelberg, Germany, 2015; pp. 133–177.
27. Zhang JJ, Fu MC, Zeng H, Geng YH, Hassani P. Variations in ecosystem service values and local economy in response to land use: A case study of Wu'an, China. Land Degrad. Dev. 2013; 24(3): 236–249.
28. Xu ZJ, Feng JF, Cheng P, Zhang Y. Estimatioin of ecological storage and its comprehensive response evaluation to land use transformation in coal mining area. Transactions of the Chinese Society of Agricultural Engineering. 2018; 34(12): 258–266. (In Chinese)
29. Wang LJ, Zhang SL, Yin PC, Wang LC, Peng SG. Spillover Effect of the Artificial Wetland in the Coal Mining Subsidence Lake on Surrounding Land Price: A Case Study on Jiuli Lake Wetland in Xuzhou City. Scientia Geographica Sinica. 2017; 37(8): 1234–1242. (In Chinese)
30. Gutjahr A. Geostatistics for sampling designs and analysis. ACS Symposium Series 1991; 465: 48–90.
31. Meng QM, Liu ZJ, Borders BE. Assessment of regression kriging for spatial interpolation – comparisons of seven GIS interpolation methods. Cartogr. Geogr. Inf. Sci. 2013; 40(1): 28–39.
32. Dai WT, Dong JH, Yan WL, Xu JR. Study on each phase characteristics of the whole coal life cycle and their ecological risk assessment: a case of coal in China. Environ. Sci. Pollut. Res. 2017; 24(2): 1296–1305.
33. Wang J, Wang R, Zhu Y, Li J. Life cycle assessment and environmental cost accounting of coal-fired power generation in China. Energy Policy. 2018; 115: 374–384.
34. Zheng MN, Deng KZ, Fan HD, Du S. Monitoring and Analysis of Surface Deformation in Mining Area Based on InSAR and GRACE. Remote Sensing. 2018; 10: 1392.
35. Xu ZJ, Zhang Y, Yang AO, Liu FW, Bi RT, Zhu HF, et al. Effect of Underground Coal Mining on the Regional Soil Organic Carbon Pool in Farmland in a Mining Subsidence Area. Sustainability. 2019: 11: 4961.
36. Fang AA, Dong JH, Zhang R. Simulation of Heavy Metals Migration in Soil-Wheat System of Mining Area. Int. J. Env. Res. Pub. He. 2019; 16: 2550.
37. Feng SS, Hou W, Chang J. Changing Coal Mining Brownfields into Green Infrastructure Based on Ecological Potential Assessment in Xuzhou, Eastern China. Sustainability. 2019; 11: 2252.
38. Hou HP, Zhang SL, Ding ZY, Huang AP, Tian YF. Spatiotemporal dynamics of carbon storage in terrestrial ecosystem vegetation in the Xuzhou coal mining area, China. Environ. Earth Sci. 2015; 74(2): 1657–1669.
39. Huang J, Wang P, Xu CR, Zhu ZZ. Fly Ash Modified Coalmine Solid Wastes for Stabilization of Trace Metals in Mining Damaged Land Reclamation: A Case Study in Xuzhou Coalmine Area. Int. J. Env. Res. Pub. He. 2018; 15: 2317.
40. Hu TH, Chang J, Liu XX , Feng SS. Integrated methods for determining restoration priorities of coal mining subsidence areas based on green infrastructure: A case study in the Xuzhou urban area, of China. Ecol. Indic. 2018; 94: 164–174.
41. Dong JH, Yu M, Bian ZF, Wang Y, Di CL. Geostatistical analyses of heavy metal distribution in reclaimed mine land in Xuzhou, China. Environ. Earth Sci. 2011; 62(1): 127–137.
42. Ungaro F, Staffilani F, Tarocco P. Assessing and mapping topsoil organic carbon stock at regional scale: A scorpan kriging approach conditional on soil map delineations and land use. Land Degrad. Dev. 2010; 21(6): 565–581.
43. Xie GD, Zhang CX, Zhen L, Zhang LM. Dynamic changes in the value of China’s ecosystem services. Ecosyst Serv. 2017; 26: 146–154.
44. Liu Y, Li JC, Zhang H. An ecosystem service valuation of land use change in Taiyuan City, China. Ecol. Model. 2012; 225: 127–132.
45. Xie GD, Liu JY, Xu J, Xiao Y, Zhen L, Zhang, CS, et al. A spatio-temporal delineation of trans-boundary ecosystem service flows from Inner Mongolia. Environ. Res. Lett. 2019; 14(6): 065002.
46. Leggett CG, Bockstael NE. Evidence of the effects of water quality on residential land prices. J. Environ. Econ. Manage. 2000; 39(2): 121–144.
47. Yu JH, Zhang WZ, Dong GP. Spatial heterogeneity in the attributes prices of residential land in Beijing. Geogr. Res. 2013; 32(6): 1113–1120. (In Chinese)
48. Kanasugi H, Ushijima K. The impact of a high-speed railway on residential land prices. Pap. Reg. Sci. 2018; 97: 1305–1335.
