# Supplementary material for: Assessing the ecological regime and spatial spillover effects of a reclaimed mining subsided lake: A case study of the Pan'an Lake wetland in Xuzhou
Source: PLoS One. 2020 Aug 27;15(8):e0238243. doi: 10.1371/journal.pone.0238243 (PMC7451651; doi:10.1371/journal.pone.0238243)
Supplement: Attachment — Submitted filename: Review_Assessing the ecological regime and its spatial spillover effect.docx [file pone.0238243.s004.docx]

Introduction: At the end of the Introduction I would suggest to state more clearly your assumptions, i.e. “ we assumed that there are spillover effects on the surrounding area of the lake and benefits downstream the ecological restoration compared to the unrestored conditions that are typically unaccounted in …” and then describe the ms content, as you did.

I would suggest a careful read through the paper to correct typos and improve the English flow. I noted a few suggestions below for the first part of the Introduction.

Line 45: “is” instead of “are”

Line 49: use the present tense “inevitably destroy”

Line 52: it doesn’t seem to be a rate, but just a quantity

Line 53 I would use the International System of Units with m^2^

Line 56: I don’t think it’s correct to use the term “sustainable development” for coal mining activities. Therefore, I would write instead: “in the current studies on the effect motigiation of coal mining”.

Line 57: remove “scientifically”

Line 60: It’s not the water table to be shallow but the unsaturated zone: “Because the unsaturated zone…”

Line 61: “expands due to sustained coal mining “

Line 64: I would remove “as the balance of water system is broken”: “as the water body may be polluted…”

Line 66: “These places have very little land-use value for agricultural or urban development”

Line 67: I don’t agree very much with this sentence, because land reclamation is a rectifying measure and not a minimizing measure: in fact, you are not limiting the implementation of the coal mining. Therefore, I would express the concept of the importance of land reclamation in a different way.

Line 70: Rephrase it, maybe: “In recent years, there has been an attempt to explore the ecological restoration model of converting mining subsidence areas into artificial wetland parks according to local conditions.”

Line 76: make the sentence simpler.

Line 79: “Wetlands offer many essential ecosystem services to human being, such as climatic regulation, food (?) supply, water purification, entertainment and biodiversity”.

Line 82: “Wang et al. [17] constructed an ecological evaluation system for wetland reclamation, which is composed of the values of products, regulation services and cultural services of the ecosystem, and evaluated the changes of their total amount in the Nansi Lake Wetland ecosystem by using remote sensing image data from 1985”.

Line 81-116: have another read and improve written English.

Line 100: focused

Line 121-124: I think that the message is obscured. It’s not uncommon that rural areas have lower economic performances than nearby urban areas; therefore, it’s not very insightful to compare directly Jiawang area and Xuzhou City.

Line 126: “from ecological restoration”

M&M: this section is too short and many details are omitted, so that it would be very difficult to reproduce the methods independently. No reference is given for the software you used, and, most importantly, the three models are very poorly described (see my comments below). In addition, some methods relevant to the land prices are moved to the Results section, creating much confusion.

Line 135: you can use numbering to indicate the different data sources, such as i) QuickBird high resolution images; ii) 1:10,000 topographic map…etc.

Line 135: I’ve never used QuickBird but I found some information that it decayed in 2015. Could you provide more info in the paper about QuickBird satellite?

Line 139: please provide the source, version, etc of ERDAS and Random Forest software.

Line 142: these are 7 soil use types, not 6!

Line 144: I guess it is the Cohen’s kappa coefficient. You need to add in the paper which accuracy requirements you set and why and/or you can present the coefficient values in the supporting information.

Line 158: here and elsewhere you can better write: “The model proposed by Costanza et al. to evaluate the ecosystem services …”. From this paragraph it is not clear how you calculated the ecosystem service coefficients, which is very important to support your model. Only in the Discussion section I understood that the ecosystem service value coefficients were taken from Costanza and modified. You should report exactly what you did for each ecosystem (or land use type) and report the ecosystem service values in Table 2 with some motivation. Please, check the units between Table 1 and Table 2.

Line 185: It’s very hard from this paragraph to understand the meaning of the ecological storage concept –and its difference from the ecosystem services –. Moreover, no indication is given for the ecological service values. Please, expand.

Line 215: here it is not explained how you consider the surrounding area of the lake; in fact, the explanation is given in the Results section. Please, move all the explanations here. I see that the formula proposed has a double summation, but in reality you are using only the residential land price, ending up with a single summation. This should be clearly stated here: the formula in Line 215 is general, but then you apply a simplified version and you should explain why. Please, explain what you use for interpolating the data and present the grid.

Results and Analysis: provide more analysis and ore details on the ecological storage value. The part on the sillover effect should be improved.

Line 224: “are shown”

Line 242: it is Table 4. Line 247: please-write this sentence.

Line 263: these values in RMB do not appear in Table 5, where the ecological storage state is presented instead. Since the whole concept of ecological storage was poorly presented in M&M without describing what it means in terms of (maybe?) ecological diversity, landscape diversity, etc. in each land use type (or ecosystem), it is very hard to appreciate the results of this paragraph. This part has to be extended in M&M and Results.

Line 282-312: most of what you write here should be in the M&M, please move it there. Why did you chose 47 samples in 2008 and 61 in 2017? How many residential land price points did you replace and please explain better why?

Line 288: could you provide some numbers for the statistical tests?

Line 309: how is *ALP* related to *ri* from line 215? You should be consistent with the nomenclature introduced in Line 215, here there is some confusion in its application.

Discussion: the importance of the study is not emphasized in this session: which aplications it opens up, which restoration measures have mostly contributed to the given results, why ecological vlue should be accounted for, etc. You should really improve it. The paragraph on uncertainty analysis cannot be appreciated because most of the methodological details are not provided in the M&M.

Line 407: these numbers have not been reported previously and should not be in the Conclusions sections, but in the Results section.
