# Supplementary material for: Assessing the ecological regime and spatial spillover effects of a reclaimed mining subsided lake: A case study of the Pan'an Lake wetland in Xuzhou
Source: PLoS One. 2020 Aug 27;15(8):e0238243. doi: 10.1371/journal.pone.0238243 (PMC7451651; doi:10.1371/journal.pone.0238243)
Supplement: Attachment — Submitted filename: Reviewer comment.docx [file pone.0238243.s003.docx]

This manuscript selected a representative reclaimed wetland park in Xuzhou city to assess the ecologic regime change and spillover effect, the results demonstrated that the ecosystem service value and ecological storage capacity have changed after the subsided waterbody changed to wetland, which will help improve the land price of surrounding areas.

I am excited to see an example study of land subsidence reclamation. However, I have several concerns that need carefully review and improvement before publication.

**Writing and grammar:**

The manuscript needs a thoroughly writing editing.

1. The writing of this manuscript is hard to understand, to name a few,

Line 23: “has been attempted in China”, and Line 70 “an attempt to”

Line 27: “wetland **derived from**”

Line 111:”**With** …”

Line 131: “had been **completed**”

The wording and grammar need more carful checking.

2. I found the manuscript maybe was from a dissertation, for there are two words of “dissertation” showed in Line 156 and 387, which should be revised.
3. There are some spelling mistakes in the manuscript, such as Line 238 “landscape”, Line 330 “calculation”.

**Name unifying:**

4. In Abstract section, “eastern China” was used to locate the study area, while in Line 59, it turned into “North China”, please unify the diction. Generally, we will use north China or North China Plain to indicate location of the high water table mining area.
5. In Table 3, you used “Urban/ Industry/Mining area”, while you named it “urban/rural construction land and industrial/mining construction land” in Line 144.
6. In Table 4, “Natural Waterbody”, while you used “general water bodies” in Line 143.

**Figures and Tables:**

7. Figure 1. There is need to put a compass in the figure.
8. Figure 1. From my view, the redline areas is not just a wetland, it is the scope of a mining region.
9. Figure 2. Upper right, what is the meaning of “Planned land”?
